# Fragmented geographies and trauma pathways in the Middle East

**DOI:** 10.1136/bmjgh-2025-021217

**Published:** 2026-05-04

**Authors:** Mac Skelton, Ghassan Abu Sittah, Zahi Abdul Sater, Anas Ismail, Hannah B Wild, Rasheed M Fakhri, Gemma Bowsher, Richard Sullivan, Craig Jones

**Affiliations:** 1 Institute of Regional & International Studies, American University of Iraq, Sulaimani, Iraq; 2 Centre for Conflict and Health Security, King’s College London, London, UK; 3 Global Health Institute, American University of Beirut, Beirut, Lebanon; 4 College of Public Health, Phoenicia University, Zahrani, Lebanon; 5 Population Health Sciences Institute, Newcastle University, Newcastle upon Tyne, UK; 6 School of Geography, Politics and Sociology, Newcastle University, Newcastle upon Tyne, UK; 7 University of Washington, Seattle, Washington, USA; 8 Médecins Sans Frontières, Paris, France; 9 Institute of Cancer Policy, Kings College London, London, UK

**Keywords:** Global Health, Health systems, Traumatology, Interdisciplinary Research, Violence

## Abstract

Enduring and interlocking conflicts across the Middle East over the last 25 years have generated injuries on a large scale. While civilian injuries in the region have been widely documented, very little is known about civilian trauma pathways—the system designed to manage patients from the moment of injury through to rehabilitation. Military trauma pathways are premised on rapid evacuation, coordinated referral and timely access to treatment, but replicating these systems for civilians, under conditions of active and ongoing conflict, presents many challenges. Civilians can be injured almost anywhere—far away from hospitals, without access to ambulances, emergency and other services—and in absence of a clear trauma pathway, their care trajectory in the weeks and years after sustaining injury is highly inequitable and geographically fragmented. This analysis examines some of the problems posed by the geographical fragmentation of civilian trauma pathways and reflects on what might be done to rebuild and improve them.

**SUMMARY BOX**

- While civilian injuries in the the Middle East have been widely documented, very little is known about civilian trauma pathways.
- Civilians can be injured almost anywhere—far away from hospitals, without access to ambulances, emergency and other services—and in the absence of a clear trauma pathway, their care trajectory in the weeks and years after sustaining injury is highly inequitable and geographically fragmented.
- This analysis examines some of the problems posed by the geographical fragmentation of civilian trauma pathways and reflects on what might be done to rebuild and improve them.

## Fragmented geographies and trauma pathways in the Middle East

 Enduring and interlocking conflicts across the Middle East over the last 20 years have generated injuries on a large scale. While civilian injuries in the region have been widely documented,^1^,^3^ very little is known about civilian trauma pathways—the system designed to manage patients from the moment of injury through to rehabilitation.^4 5^ Military trauma pathways are premised on rapid evacuation, coordinated referral and timely access to treatment,^6^ but replicating these systems for civilians, under conditions of active and ongoing conflict, presents many challenges.^7^ While militaries operate within defined geographical areas and with clear trauma pathways, civilian trauma pathways are much more fragmented. Civilians can be injured almost anywhere—far away from hospitals, without access to ambulances, emergency and other services^8 9^—and in the absence of a clear trauma pathway, their care trajectory in the weeks and years after sustaining injury is highly inequitable and geographically fragmented.

Civilians injured across the Middle East must contend with often-opaque and difficult-to-navigate trauma pathways. Some civilians are able to access care locally, while others seek care across international borders.^10^ This analysis examines some of the problems posed by the geographical fragmentation of trauma pathways and reflects on what might be done to rebuild and improve them.

## The regional burden of conflict-related injury

The conflict in Gaza since 7 October 2023 has resulted in more than 72 200 fatalities and over 171 800 injuries as of 16 March 2026.^11^ Recent studies estimating mortality from traumatic injury after 9 months^12^ and after 15 months^13^ have suggested that the figures provided by the Gaza Ministry of Health under-report by 41% and 34.7%, respectively. According to a WHO estimate in September 2024, approximately 25% of those who have been injured in Gaza will have acute and ongoing rehabilitation needs—with approximately 15 000 extremity injuries, 3000–4000 amputations, over 2000 major head and spinal cord injuries and over 2000 casualties with major burns.^14^ Other conflicts in the region have also generated significant injury burdens. Israel’s military operation in Lebanon in October/November 2024 resulted in approximately 4047 civilian deaths and 16 638 injuries.^15^ In Syria, 14.9% of households reported at least one violence-related disability, annually, between 2011 and 2020.^16^ Between 2011 and 2016, over a quarter of barrel bomb injuries among civilians were children, with a quadrupling in the number of conflict-related deaths among women.^17^ During the first decade of conflict in Baghdad since 2003, 39% of conflict-related injuries resulted in death and 56% resulted in life-changing disability.^1^ At the time of writing (March 2026), a major conflict between the US and Israel and Iran has spread across the Middle East which will only add to the regions’ already significant burden ofinjury.^18^

A high burden of injury characterised by successive mass casualty events can present challenges even in well-resourced healthcare systems during peacetime.^19^ These challenges are significantly exacerbated across the Middle East not only because violent conflict is ongoing but also because conflict has directly and indirectly damaged, destroyed and otherwise incapacitated care provision, including the destruction of hospitals and killing of healthcare workers.^20^,^22^ Previously functional and effective national health systems have been degraded through conflict, sanctions, siege and other forms of insecurity such that healthcare systems are significantly under-resourced and understaffed precisely at the time they most needed.^23 24^

## Conflict injuries ‘on the move’

New regional geographies of care have emerged across the region in response to conflict. This regional geography is characterised by mass displacement and migration, referral of patients out-of-country and by civilians seeking safety, care and better life and livelihoods abroad.^25 26^ During the US-led invasion and occupation of Iraq (2003–2011), as well as the ISIS conflict of 2014–2017, injured Iraqis travelled to private hospitals in Lebanon and Jordan for advanced surgical care.^25^ In Gaza, a small percentage of injured Palestinians have been granted travel permission to neighbouring countries^27^ including Egypt, UAE, Qatar, Turkey, Jordan, Lebanon and Iraq as well as to European countries,^28^ but the WHO says a further 14 000 require medical evacuation from Gaza.^29^ Similar cross-border dynamics have been documented in the wake of the conflict in Libya.^30^ These cross-border geographies make managing and tracking patients across the trauma pathway even more challenging.

## Right to health: inequities and barriers

Inequitable access, political violence and restrictions on movement constitute significant barriers to the right to health during armed conflict.^31^ Access to healthcare and health outcomes are determined not only by injury patterns but also by a broad range of factors including demographic characteristics, politics and geography.^32^ Being a soldier versus being a civilian can mean the difference between receiving care across a highly organised and well-resourced trauma pathway, including forward deployed emergency medical teams and casualty evacuation protocols and having to improvise one’s own care in informal and fragmented ways.^33 34^ The 'golden hour' after sustaining injury described for military casualties rarely exists for civilian conflict casualties.^35^ Each checkpoint, permit denial or other impediment to mobility elongates the time from point of injury to reaching surgical care, with unquantified implications for preventable death and disability.^36^ Ongoing research by authors Skelton and Jones on civilians injured in the 2016–2017 Battle for Mosul shows that place and time of injury, residence location and educational status—among many other factors—have significant bearing on access to and quality of care across the trauma pathway. These disparities along the trauma pathway raise fundamental questions of equity and the right to health under both international humanitarian law and international human rights law.

Under conflict conditions, triage can be governed by procedures that are opaque and highly politicised.^37^ In Gaza, the vast majority of patients requiring care abroad have been prevented from leaving, with some exceptions made for reasons that are neither transparent nor well understood.^27^ According to the Norwegian Refugee Council, restrictions imposed on Yemen’s airspace by the Saudi-led military coalition have blocked the medical evacuation of at least 32 000 Yemeni patients in need of life-saving treatment.^38^ Restrictions on mobility and access to healthcare occur within as well as across borders.^39 40^ Such restrictions lead to significant supply issues, including essential medical supplies and even amputations conducted without anaesthesia.^41 42^ Spatial fragmentation in acute care inevitably resurface years later as increased demand for revision surgery, mobility aids and psychosocial support.^1 43^

## Rebuilding trauma pathways

The spatial fragmentation of trauma pathways across the Middle East will persist long after specific conflicts end. Narrow and local clinical interventions and innovations are vital but insufficient. Any sustainable solution must grapple with the difficult political realities of health financing because trauma pathways must be resourced. Understanding the centrality of patient mobility is an essential first step in creating a regional fund to support patients on their journeys. At present the burden falls disproportionately on patients and their families.^30 44^ Despite the enormous financial burden on families, cross-border care and fragmentation will thrive so long as specialist care is not available locally, emphasising the importance of equipping and reintegrating specialists into local public health systems as well as encouraging the return and integration of qualified specialists in diaspora.^45^ The WHO’s Regional Trauma Initiative, established in the Eastern Mediterranean in 2020 offers a promising model in providing technical and operational assistance and capacity-building and should be a priority for financing. The Initiative has supported the response to more than 200 mass casualty incidents across five conflict-affected settings with an estimated reach of 800 000 injured patients^46^

The killing and displacement of doctors and healthcare workers across the region remains a serious challenge and any sustainable solution must include the rebuilding and training of human resources for health. Successful programmes have already demonstrated the viability of such approaches, including the training of hundreds of Palestinian specialists in Qatar.^47^ Such partnerships are an excellent way to decrease dependence on cross-border healthcare and provide one example of building sustainable trauma pathway solutions even in a region where conflict is once again afoot.

The spatially fragmented trauma pathways described in this commentary represent not only a clinical challenge but a systemic vulnerability to health governance across the Middle East. These complex dynamics of injury and mobility demand coordinated regional action grounded in equity and the right to health. Without sustained commitment and dedicated financing, the cycle of injury and poor health outcomes will continue to deepen across the Middle East.

## Reflexivity statement

The authors are clinicians and social scientists who have worked for many years in conflict settings across the Middle East, including Iraq, Syria, Lebanon and Palestine. Our engagement with this topic emerges from a combination of clinical collaboration, ethnographic research and policy work with local health professionals, humanitarian actors and patients navigating trauma care during and after conflict.

## Data Availability

There are no data in this work.
